# Herbal medicine and acupuncture for mild cognitive impairment: a retrospective study of 2,242 for older adults in Republic of Korea

**DOI:** 10.3389/fneur.2025.1628794

**Published:** 2025-10-29

**Authors:** Hyo-Weon Suh, Jae Hwan Lim, Jin-woo Suh, Chan Park, Yong-Sin Park, Sun-Yong Chung

**Affiliations:** ^1^Clinical Evidence Research Team, Division of Healthcare Research, National Evidence-based Healthcare Collaborating Agency, Seoul, Republic of Korea; ^2^Seoul Korean Medicine Association, Seoul, Republic of Korea; ^3^Wirye Kyunghee Korean Medicine Clinic, Seongnam, Republic of Korea; ^4^Department of Korean Neuropsychiatry, College of Korean Medicine, Sangji University, Wonju, Republic of Korea; ^5^Department of Clinical Korean Medicine, Graduate School, Kyung Hee University, Seoul, Republic of Korea; ^6^Balkeunnun Korean Medicine Clinic, Seoul, Republic of Korea; ^7^Department of Neuropsychiatry, Kyung Hee University Korean Medicine Hospital at Gangdong, Seoul, Republic of Korea

**Keywords:** cognitive dysfunction, mild cognitive impairment, herbal medicine, acupuncture therapy, propensity score

## Abstract

**Introduction:**

Dementia prevalence increases with age, underscoring the importance of early intervention for mild cognitive impairment (MCI). However, standard treatment recommendations for MCI remain lacking. Herbal medicine and acupuncture have been proposed as potential alternatives. This study evaluated the feasibility and effectiveness of these interventions in patients with MCI.

**Methods:**

We conducted a retrospective cohort study using data from a public health promotion program for community-dwelling older adults in Korea who received acupuncture, with or without herbal medicine, between 2021 and 2023. Feasibility was assessed by completion of acupuncture sessions and follow-up. Exact propensity score matching was performed using age, sex, comorbidities, depression scores, and health-related behaviors to compare herbal medicine add-on versus acupuncture only groups. Clinical outcomes included cognitive function [the Cognitive Impairment Screening Test (CIST) and the Montreal Cognitive Assessment (MoCA)] and depression [Geriatric Depression Scale–Short Form (GDS-SF)].

**Results:**

Of 5,525 participants, 4,623 received acupuncture with or without herbal medicine. Feasibility was high, with 86.4% completing planned acupuncture sessions; among these, 93.8% also received herbal medicine. Loss-to-follow-up rate was lower in the herbal medicine add-on (4.1%) than in the acupuncture-only (12.1%). After matching, 2,242 participants were included (2,044 herbal medicine add-on and 198 acupuncture-only). Both groups showed significant improvements in CIST, MoCA, and GDS-SF scores, with the herbal medicine add-on group demonstrating significantly greater cognitive improvement in the CIST (coefficient: 0.58; 95% CI, 0.10–1.08).

**Conclusion:**

Herbal medicine combined with acupuncture appeared feasible and potentially effective for managing MCI, supporting its practicality in community settings. However, its therapeutic benefits need to be further validated through rigorously designed randomized controlled trials. Long-term studies are warranted to confirm these findings and clarify their role in dementia prevention.

## 1 Introduction

The prevalence and burden of dementia are rapidly increasing with the aging global population. By 2050, more than two billion people (22% of the global population) will be aged ≥ 60 years (1), and the number of individuals living with dementia is projected to reach 139 million (2). The associated economic costs, estimated at US $1.3 trillion in 2019, are expected to rise substantially, underscoring the urgent need for early diagnosis and preventive interventions (2).

Mild cognitive impairment (MCI), often considered a prodromal stage of dementia, is defined as measurable cognitive decline with largely preserved daily functioning (3). MCI affects about 22% of adults aged ≥ 65 years (4), and carries a clinically significant risk of progression to Alzheimer’s disease, with reported annual conversion rates ranging from 14 to 87%, depending on study design and diagnostic criteria (5).

Although early intervention for MCI is of major importance to public health, therapeutic options remain limited (6). The 2022 American Academy of Neurology guidelines concluded that evidence is insufficient for pharmacological therapies and recommended nonpharmacological strategies such as exercise and cognitive training (7). However, adherence to these strategies is often poor in older adults because of limited awareness of MCI, comorbid conditions, low social support, and restricted accessibility (8, 9).

Consequently, traditional medicine approaches have attracted considerable attention. Systematic reviews have suggested that herbal medicine (10) and acupuncture (11) may improve cognitive function in patients with MCI. However, most trials were small, heterogeneous, and of limited generalizability.

To address these gaps, we conducted a retrospective cohort study to evaluate the feasibility and effectiveness of an 8–10-week combined intervention of herbal medicine and acupuncture in a local government–initiated dementia prevention program for community-dwelling older adults with MCI, thereby generating real-world evidence for the potential integration of traditional medicine into public health strategies.

## 2 Materials and methods

### 2.1 Data sources

We used the Korean Medicine Senior Health Promotion Program (KSHPP) cohort, which includes participants aged 60 years and older who utilized Korean medicine public health services in Seoul, Republic of Korea. The KSHPP was conducted in 2017. In this study, we retrospectively analyzed KSHPP cohort data from 2021 to 2023. During the program, the KSHPP provides acupuncture and/or herbal treatments (decoction or granules) to elderly individuals at high risk of MCI. The study protocol was approved by the Institutional Review Board of Kyung Hee University, Korea (KHSIRB-23-418[EA]).

The overall KSHPP process and outcome measurements are shown in Supplementary Figure 1. Informed consent was obtained from all the participants at the start of the program.

### 2.2 Subjects

The inclusion criteria were as follows: (1) participants in the KSHPP between 2021 and 2023; (2) aged 60 years or older; (3) Montreal Cognitive Assessment (MoCA) score <23 at baseline; (4) those who received acupuncture only or combined treatment of herbal decoction and acupuncture; (5) received acupuncture treatment from 12 to 20 sessions; and (6) followed the standard treatment protocol. The subjects were retrospectively divided into two groups: the herbal medicine add-on group (acupuncture and herbal medicine) and the acupuncture-only group. For analysis, we excluded (1) those who did not have available pre- and post-treatment MoCA, Cognitive Impairment Screening Test (CIST), and Geriatric Depression Scale-Short Form (GDS-SF) scores and (2) those with no matching subjects between the two groups.

### 2.3 Acupuncture

The acupuncture protocol was standardized. Treatment acupoints were GV20, EX-HN1, LI4, LR3, ST36, HT7, and PC6. The duration of acupuncture was 25 min. The recommended number of acupuncture sessions was 12–20 (twice weekly).

### 2.4 Herbal medicine

Each participant received one of eight herbal medicines as a decoction for 15 days. These herbal medicines are multi-herbal formulas as follows: *Guibi-tang* (12 herbs), *Modified Guibi-tang* (14 herbs), *Yukmijihwang-tang* (6 herbs), *Cheonwangbosim-dan* (15 herbs), *Jowiseungcheong-tang* (14 herbs), *Ondam-tang* (8 herbs), *Modified Ukgan-san* (9 herbs), and *Hwanglyeonhaedok-tang* (4 herbs). The detailed compositions of these herbal formulas are summarized in Supplementary Table 1. The selection of a specific herbal formula for each participant was determined by a doctor of Korean medicine according to the principles of pattern identification.

### 2.5 Outcome measurements

Feasibility was primarily assessed by the proportion of participants who completed acupuncture treatment, which was the mandatory component of the program. In addition, we evaluated the proportion of participants who additionally received herbal medicine and follow-up completion rates, stratified by treatment group.

Clinical outcomes were assessed as secondary objectives. To observe the effectiveness of the KSHPP using herbal medicine and acupuncture, we assessed improvement rate and the pre-post changes in the CIST, MoCA, and GDS-SF scores.

The improvement rate was calculated as the proportion of participants whose change in CIST scores exceeded the minimal clinically important difference (MCID). The MCID for the CIST was defined as 0.5 standard deviation (SD) of the normative values based on the previous review (12).

CIST is a cognitive screening test developed by a public medical institution for use in the National Dementia Screening Program in Korea (13). The CIST is free to use, and the materials can be downloaded from the website of the National Institute of Dementia in Korea. The cutoff score was presented according to age and years of education (range: 10–27 scores). A higher CIST score indicated an improvement in cognition.

The MoCA is a screening tool for MCI, rather than dementia, in older adults. The cutoff score was 26 in the original version (14); however, currently, a cutoff score of 23 is recommended for better diagnostic accuracy (15, 16). The Korean version of the MoCA has been validated in a clinical setting, and a cutoff score of 23 has been adopted (17). A higher MoCA score indicated an improvement in cognition.

The GDS-SF is used to evaluate depressive symptoms in older adults. This scale distinguishes between symptoms caused by increasing age and those caused by depression and consists of simple and easily understandable questions for older adults. The previously developed 30-item binary scale (18) was simplified to include 15 items (19), and the short form has been validated with a cutoff score of five in Korea (20). A lower GDS-SF score indicated an improvement in geriatric depression.

### 2.6 Study covariates

Age, sex, comorbidities such as hypertension, diabetes, and hyperlipidemia based on concomitant medications collected through history taking at baseline, geriatric depression assessed by the GDS-SF, and health-related behaviors such as smoking, drinking, and exercise based on self-reporting at baseline were obtained as covariates in this study. Adjustments for medications affecting cognition, such as anti-inflammatory drugs (e.g., steroids or biologics) and cholinergic agents (e.g., cholinesterase inhibitors or anticholinergics), could not be performed because the medical records primarily documented indications rather than specific drug names.

### 2.7 Statistical analysis

Patient baseline characteristics were summarized using descriptive statistics. Continuous variables were compared between the two treatment groups using either an independent samples t-test for normally distributed data or the Mann–Whitney U test for non-normally distributed data. For categorical variables, group comparisons were performed using the Chi-square test or Fisher’s exact test when appropriate.

To assess the changes between the pre- and post-treatment values within each group, the Wilcoxon signed-rank test was applied, as the data exhibited non-normality and included outliers. This non-parametric test was used to compare pre- and post-intervention differences in cognition and depression outcomes within each group.

Given the non-normality and presence of outliers in the outcome data, attributable to the chart-based nature of the data, we employed a robust regression approach (M-estimation) to further analyze the treatment effects. Robust regression was performed using the rlm function in the MASS package of R. This method provides robust estimates of regression coefficients when the data contain outliers or violate the assumptions of normality.

Owing to the significant imbalance in sample sizes between the two groups—3,852 patients in the herbal medicine add-on group and 222 patients in the acupuncture-only group—sensitivity analysis was conducted to assess the impact of this discrepancy on the results. To address the potential bias from unequal sample sizes, two propensity score matching (PSM) methods were applied: 1:1 nearest-neighbor matching and 1:n exact matching. Propensity scores were estimated using logistic regression and the participants were matched based on these scores. The standardized mean difference (SMD) was calculated for all covariates to evaluate the balance achieved through matching; an SMD of less than 20% was considered indicative of successful balancing. After matching, robust regression was reapplied to assess the treatment effects in a more balanced sample, considering weight adjustment for the exact matching method.

To enhance the robustness of our findings and explore the consistency of treatment effects, we performed two sets of additional analyses on the propensity score-matched cohort. First, within the herbal add-on group, we used robust regression model (M-estimation) to compare changes in cognitive outcomes (ΔCIST) across major herbal prescriptions. The model was adjusted for the study covariates described in Section 2.6. This analysis allowed us to investigate whether specific herbal formulas demonstrated differential efficacy. Second, we conducted pre-specified subgroup analyses using robust regression stratified by sex (male vs. female) and baseline depressive symptom risk (GDS-SF ≥ 5 vs. <5). This allowed us to examine the consistency of treatment effects across these key patient characteristics. We also fitted interaction terms between treatment allocation and subgroup in the same robust regression framework to determine if the treatment effect significantly differed between them.

The significance of the results was evaluated using t-values and confidence intervals (CIs). Statistical significance was set at *p* < 0.05. All analyses were performed using R version 4.2.3, with the MASS and MatchIt packages used for robust regression and PSM, respectively.

## 3 Results

### 3.1 Characteristics of subjects

**Figure 1:**
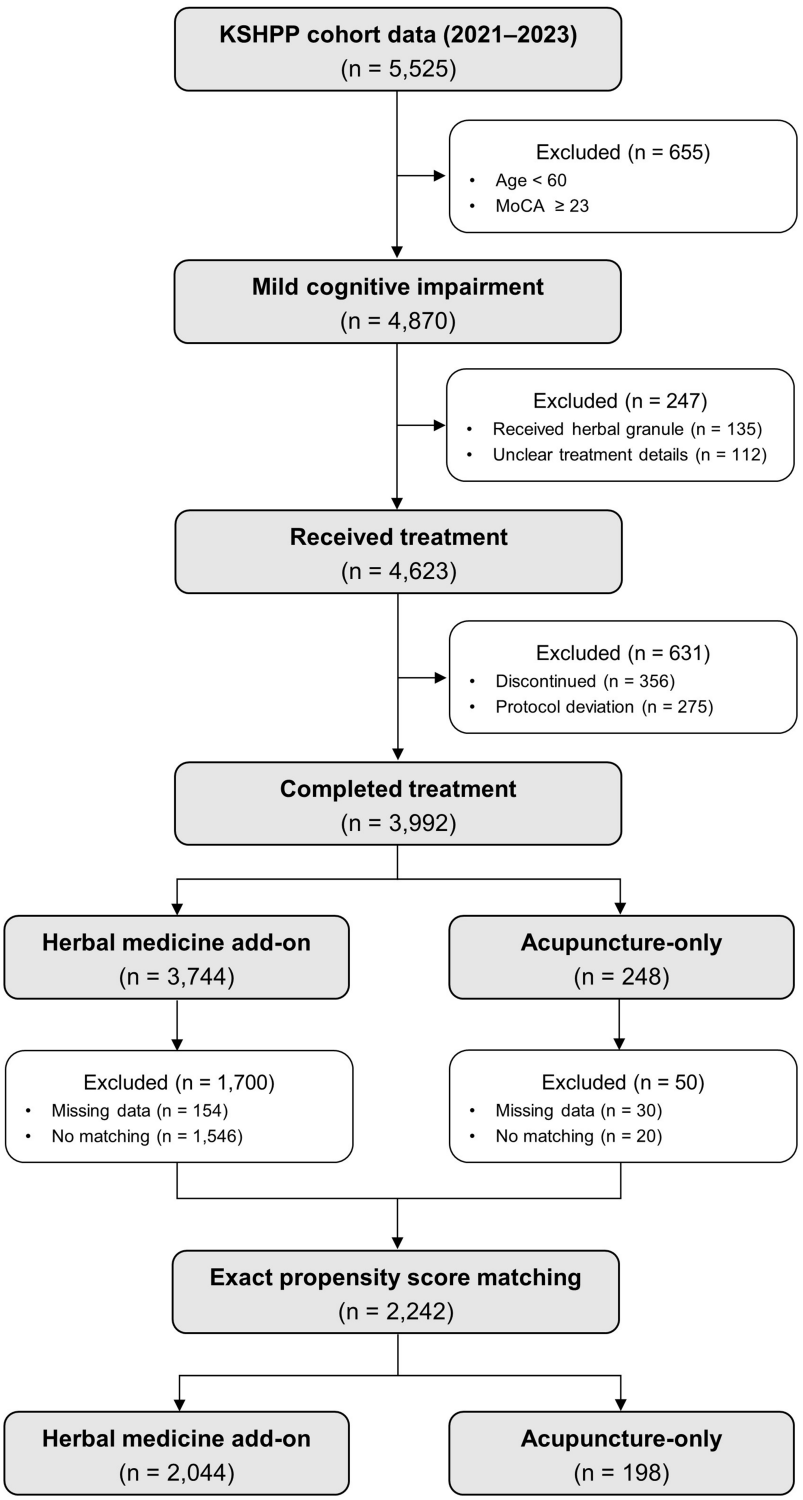
Flow chart of the study.

Of the 5,525 participants initially registered in the KSHPP cohort, 4, 870 were at risk for MCI and aged 60 years or older. Of these, 4,623 received acupuncture treatment, and 3,992 (86.4%) completed the treatment. After matching, we obtained a matched sample of 2,242 patients, consisting of 2,044 herbal medicine add-on users and 198 acupuncture-only users, for the final analysis (Figure 1). As adjusted SMD of each outcome variable was smaller than 0.2, baseline outcome balance after matching was confirmed. Table 1 presents the baseline characteristics of the two groups.

**Table 1:** Baseline characteristics after exact propensity score matching.

Characteristics	Herbal medicine add-on (*n* = 2,044)	Acupuncture- only (*n* = 198)
Demographic characteristics		
Age [year, median (IQR)]	74.0 [71.0, 78.0]	75.0 [71.0, 80.0]
Women (*n*, %)	1,884 (92.2)	157 (79.3)
Education [year, median (IQR)]	7.0 [6.0, 12.0]	7.0 [6.0, 12.0]
Clinical characteristics		
Comorbidities (*n*, %)^a^	1,414 (69.2)	158 (79.8)
Hypertension (*n*, %)	1,043 (51.0)	122 (61.6)
Diabetes mellitus (*n*, %)	328 (16.0)	63 (31.8)
Hyperlipidemia (*n*, %)	582 (28.5)	66 (33.3)
Geriatric depression (*n*, %)^b^	1,433 (70.1)	131 (66.2)
Health-related behaviors^c^		
Smoking (*n*, %)	0 (0)	0 (0)
Drinking (*n*, %)	88 (4.3)	21 (10.6)
Exercise (*n*, %)	1,501 (73.4)	135 (68.2)
Cognitive function and depression		
CIST [median (IQR)]	19.0 [16.0, 23.0]	18.0 [15.0, 22.0]
MoCA [median (IQR)]	19.0 [16.0, 21.0]	17.0 [15.0, 20.0]
GDS-SF [median (IQR)]	7.0 [4.0, 10.0]	6.0 [3.0, 9.0]

CIST, Cognitive Impairment Screening Test; GDS-SF, Geriatric Depression Scale-Short Form; IQR, interquartile range; MoCA, Montreal Cognitive Assessment.

a Comorbidities were identified based on concomitant medications collected through history taking at baseline.

b Geriatric depression was assessed using the Geriatric Depression Scale-Short Form (GDS-SF) at baseline. A GDS-SF score of 5 or higher was considered indicative of risk for geriatric depression.

c Health-related behaviors were presented based on self-reporting at baseline.

### 3.2 Feasibility

Because acupuncture was a mandatory component of the program, feasibility was primarily evaluated by completion of the acupuncture sessions. Among the 4,623 participants who initiated acupuncture, 86.4% (3,992/4,623) completed 12–20 sessions. Of these, 93.8% (3,744/3,992) also received herbal medicine. The loss-to-follow-up rate was 4.1% in the herbal medicine add-on group and 12.1% in the acupuncture-only group.

### 3.3 Cognitive function and depression before and after treatment

The improvement rates were 74.2% in the herbal medicine add-on group and 69.2% in the acupuncture-only group. Scores on the CIST, MoCA, and GDS-SF significantly improved from baseline to post-treatment in both groups (all *p* < 0.05). However, when comparing the change in each variable between groups, a significant difference was found only in the CIST (*p* < 0.05). These results are presented in Table 2.

**Table 2:** Changes in cognition and depression outcome after exact propensity score matching.

Outcomes	Herbal medicine add-on (*n* = 2,044)	Acupuncture- only (*n* = 198)
Improvement (*n*, %)	1,516 (74.2%)	137 (69.2%)
CIST (mean, SD)		
Pre	19.1 ± 4.7	18.4 ± 4.9
Post	23.2 ± 4.4^a^	21.9 ± 4.5^a^
Change	4.0 ± 3.7 ^b^	3.4 ± 3.9
MoCA (mean, SD)		
Pre	17.7 ± 3.7	17.0 ± 3.9
Post	21.4 ± 4.4^a^	20.6 ± 4.7^a^
Change	3.7 ± 3.4	3.6 ± 3.6
GDS-SF (mean, SD)		
Pre	6.7 ± 3.9	6.3 ± 3.8
Post	5.3 ± 3.7^a^	5.2 ± 3.9^a^
Change	−1.5 ± 3.0	−1.1 ± 2.5

CIST, Cognitive Impairment Screening Test; GDS-SF, Geriatric Depression Scale-Short Form; MoCA, Montreal Cognitive Assessment; SD, standard deviation.

a Within-group difference comparing pre- and post-values; Wilcoxon signed rank test, *p* < 0.05.

b Between-group difference comparing change from baseline values in each group; Mann Whitney U test, *p* < 0.05.

### 3.4 Add-on effect of herbal medicine between groups

The use of herbal medicine was associated with cognition improvement measured by CIST (*p* < 0.05; coefficients of 0.58 [95% CI, 0.10 to 1.08]) (Table 3). Regarding different herbal medicine formulas, robust regression showed no statistically significant differences in changes in cognitive scores (ΔCIST) across major herbal prescriptions after adjusting for study covariates within the herbal add-on group. Compared with *Guibi-tang* as the reference group, other formulas such as *Ondam-tang* (coefficient 0.23 [95% CI, −0.50 to 0.95]) and *Modified Ukgan-san* (0.75 [95% CI, −0.19 to 1.69]) showed numerically higher scores, but these differences were not statistically significant (Supplementary Table 2).

**Table 3:** Robust regression analysis between two groups after exact propensity score matching.

Outcomes	Coefficients	Std. error	*t* value	95% CI
CIST	0.58	0.249	2.362	(0.10, 1.08)
MoCA	0.20	0.237	0.863	(−0.26, 0.67)
GDS-SF	0.10	0.201	0.469	(−0.30, 0.49)

CIST, Cognitive Impairment Screening Test; GDS-SF, Geriatric Depression Scale-Short Form; MoCA, Montreal Cognitive Assessment.

The results of the robust regression analysis showed that the herbal medicine add-on group had an average increase of 0.20 in MoCA difference and an average increase of 0.10 in GDS-SF difference, neither of which was statistically significant (Table 3).

### 3.5 Subgroup analyses

**Figure 2:**
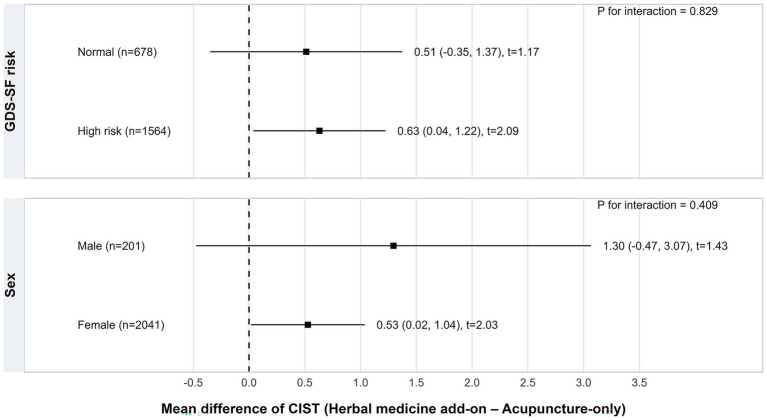
Subgroup analysis of adjusted treatment effects on CIST score change stratified by baseline GDS-SF risk and sex (exact-matched cohort). CIST, Cognitive Impairment Screening Test; GDS-SF, Geriatric Depression Scale-Short Form.

Pre-specified subgroup analyses indicated that the association between herbal medicine add-on treatment and CIST improvement was generally consistent across subgroups (Figure 2). The coefficient for CIST improvement was 0.63 (95% CI, 0.04 to 1.22) for patients at high risk of depressive symptoms and 0.51 (95% CI, −0.35 to 1.37) for those at normal risk. Similarly, coefficients were 1.30 (95% CI, −0.47 to 3.07) for males and 0.53 (95% CI, 0.02 to 1.04) for females. Tests for interaction between treatment allocation and subgroup were not statistically significant (*p* for interaction = 0.829 for GDS-SF risk and 0.409 for sex), suggesting that the treatment effect was consistent across these strata.

### 3.6 Sensitivity analyses

To assess the robustness of our findings and explore the consistency of treatment effects, we performed several additional analyses. We examined the results using 1:1 PSM with the same covariates from 422 participants (211 per group) and the association between herbal medicine use and cognitive improvement was consistent in the sensitivity tests (Supplementary Table 3). We also performed quantitative bias analyses, calculating E-values to determine the minimum strength of association an unmeasured confounder would need to have with both the treatment and the outcome to completely explain our findings. For the continuous outcome (ΔCIST), the point estimate had an E-value of 2.67, indicating that only a relatively strong unmeasured confounder could nullify the observed association.

## 4 Discussion

Although the clinical significance of managing MCI is well recognized, therapeutic options remain limited. Pharmacological interventions, such as cholinesterase inhibitors, have shown modest improvements in cognitive function but have not demonstrated efficacy in delaying or preventing progression to dementia (21, 22). Nonpharmacological strategies, including physical activity and cognitive training, are recommended (7, 23), but their effectiveness depends heavily on sustained patient engagement. This can be particularly challenging for older adults with MCI, who often experience cognitive limitations, low health literacy, or limited accessibility (8, 9).

Given this context, we examined herbal medicine and acupuncture, which are widely practiced and culturally accessible in East Asia, as alternative interventions. The herbal formulas (24–33) and acupuncture protocols (34, 35) used in this study have been previously reported to improve cognitive function. These approaches are supported by surveys of Korean medicine practitioners and are consistent with the Korean medicine clinical practice guidelines for dementia and MCI (36–38).

Systematic reviews have shown that most randomized controlled trials (RCTs) investigated either herbal medicine (39) or acupuncture (11) compared with conventional pharmacological agents such as donepezil or nimodipine. However, these two modalities are commonly combined in routine Korean medical practice. Therefore, evaluating the relative effectiveness of acupuncture alone versus combination therapy provides clinically relevant insights into real-world practice.

In this large-scale observational study, we demonstrated the feasibility and acceptability of these interventions among community-dwelling older adults with MCI. Of the 4,623 eligible participants, 86.4% (*n* = 3,992) completed acupuncture treatment, and among these, 93.8% also received herbal medicines. Importantly, the combination therapy group exhibited lower dropout rates compared with the acupuncture-only group (4.1% vs. 12.1%), indicating that integrated therapy may enhance adherence and feasibility in this population.

To address potential confounding, we conducted PSM using demographic, lifestyle, and comorbidity factors known to influence MCI progression. Previous studies have shown that advanced age and female sex increase the risk of conversion from MCI to Alzheimer’s disease, while chronic conditions such as hypertension, diabetes, and hyperlipidemia, as well as smoking and alcohol use, further accelerate decline (40, 41). Conversely, healthy behaviors—including mental activity, fruit consumption, and social engagement—promote recovery to cognitive normalcy, whereas depression predicts deterioration (42, 43). By adjusting for these covariates, we sought to provide more reliable estimates of treatment effects.

Because the CIST is a relatively new assessment tool developed in Korea in 2021, previous studies using the CIST are limited and have not established MCID. Thus, we defined “improvement” as an individual pre–post change exceeding 0.5 SD of the normative data, adjusted for age and education level, ranging from 0.57 to 2.41 points. Following matching, the herbal add-on group showed an average improvement of 4 points, whereas the acupuncture-only group showed an average improvement of 3.4 points. Based on our definition, 74.2% of participants in the herbal add-on group and 69.2% in the acupuncture-only group were classified as showing improvement.

Meanwhile, robust regression showed that the herbal medicine add-on group had significantly greater improvement in CIST scores (coefficient 0.58 [95% CI, 0.10–1.08]). Sensitivity analyses yielded an even larger effect size, reinforcing the robustness of the findings. The greater increase was observed in sensitivity analyses using 1:1 PSM (0.95 [95% CI, 0.30–1.60]). Our results align with prior evidence: an RCT reported that acupuncture combined with herbal medicine (Yishen granules) improved MoCA scores by 1.63 points compared with acupuncture plus placebo (44), and a systematic review showed that herbal medicine increased Mini Mental State Examination scores by 1.90 points compared with placebo (10). As noted above, it was not feasible to establish a single-point estimate for the MCID in our study. For other cognitive instruments such as the Mini Mental State Examination and MoCA, however, changes of approximately 1–2 points have been suggested as indicative of the MCID in populations with MCI (45, 46). The observed between-group difference of 0.58 points (95% CI, 0.10–1.08) falls below this suggested range. Although the upper bound of the confidence interval approached the lower limit of the MCID, the overlap indicates that the clinical significance remains uncertain. Taken together, the overall direction and magnitude of our findings are broadly consistent with prior herbal medicine trials, albeit with smaller effect sizes. The relatively short intervention period of 15 days, compared with the median 6-month duration in previous RCTs, may partly account for this difference.

Within the herbal add-on group, robust regression adjusting for study covariates revealed no statistically significant differences across major herbal prescriptions. Although *Ondam-tang* (coefficient 0.23 [95% CI, −0.50 to 0.95]) and *Modified Ukgan-san* (0.75 [95% CI, −0.19 to 1.69]) showed numerically higher improvements compared with *Guibi-tang*, these differences were not statistically significant.

We performed subgroup analyses based on sex and baseline depressive symptom risk because previous studies indicates that sex and geriatric depression may influence cognitive trajectories and responses to interventions in MCI. Epidemiological studies have shown sex differences in the prevalence, progression, and neuropathological features of MCI and dementia, with women often exhibiting higher risk of cognitive decline (47). Similarly, depressive symptoms are known to affect cognitive function, with evidence suggesting that individuals with higher depressive symptom burden may experience different cognitive outcomes compared with those at low risk (48, 49). Therefore, we explored these subgroups to assess whether the effects of the herbal add-on differed according to sex or baseline depression risk, while acknowledging that our findings are exploratory and interaction tests did not reach statistical significance.

The cognitive benefits observed in this study may be supported by several biological mechanisms of herbal medicine. MCI is thought to arise from multiple interacting mechanisms, including amyloid-*β* deposition and tau hyperphosphorylation leading to synaptic loss (50), oxidative stress, and mitochondrial dysfunction (51), deficits in neurotransmitter systems, particularly cholinergic dysfunction (52), chronic neuroinflammation (53), and reduced neurogenesis and neurotrophic support such as decreased brain-derived neurotrophic factor (54). Herbal medicines have been reported to inhibit amyloid aggregation (55), exert antioxidant effects via activation of Nrf2 (56), and regulate cholinergic neurotransmission, for example through acetylcholinesterase inhibition (57). In addition, herbal medicines can modulate neuroinflammation by regulating cytokine expression (58) and promote neurogenesis through upregulation of neurotrophic factors (59). Collectively, these multi-target actions provide plausible biological explanations for the cognitive improvements associated with herbal medicine add-on therapy observed in our study and support the clinical relevance of the findings.

These findings suggest that acupuncture and herbal medicine may contribute to delaying or attenuating cognitive decline in patients with MCI, supporting their potential role as a prevention program. South Korea is currently pursuing the “National Responsibility for Dementia” initiative through the 4th National Dementia Management Plan (2021–2025) (60), which establishes a nationwide framework for prevention, early diagnosis, and long-term care; however, preventive interventions remain limited. Our results provide preliminary evidence to consider incorporating public health promotion programs using acupuncture and herbal medicine into primary-care settings—including private clinics, public health centers, and dementia care centers—particularly for older adults (61). Integrating acupuncture and herbal medicine could help support a policy shift toward prevention-oriented dementia management.

Several limitations should be acknowledged. First, the retrospective observational design may not fully eliminate residual confounding, despite PSM adjustment for nine covariates. However, the E-value of 2.67 indicates that only an unmeasured confounder of moderate strength could explain away the observed association, suggesting that residual confounding is unlikely to substantially bias the results. Second, participants were grouped according to the treatment they received, resulting in unequal sample sizes between groups. Nevertheless, baseline balance was confirmed after exact PSM, with all outcome variables showing SMDs < 0.2. Additionally, 1:1 PSM was performed as a sensitivity analysis. Third, neither participants nor practitioners were not blinded, and cognitive outcomes were assessed by the same practitioners, which may have introduced performance and detection biases (62). Fourth, the short herbal treatment duration (15 days) and follow-up interval (8–10 weeks) contrast with prior RCTs that typically evaluated treatment over at least 2 months (10, 39). These design features may have led to underestimation of long-term benefits or, conversely, overestimation due to short-term learning effects. Future studies should adopt longer treatment durations and extended follow-up to better evaluate sustained outcomes. Lastly, in our study, although data were collected from multiple sites, site-level clustering was not explicitly modeled, and residual variation attributable to clinic-specific factors may exist. Future studies with larger sample sizes should consider multi-level modeling or generalized estimating equations to account for potential clinic-level effects. Moreover, the generalizability of our findings may be limited beyond the specific clinical settings included in this study. Differences in patient demographics, practitioner expertise, or treatment delivery across other sites could affect the observed outcomes. Therefore, caution is warranted when extrapolating these results to other populations or healthcare settings, and replication in broader and more diverse contexts is recommended.

## 5 Conclusion

In conclusion, our findings suggest that herbal medicine combined with acupuncture is a feasible and potentially effective intervention for managing MCI. High adherence and reduced dropout in the combination group highlight the practicality of this approach for community-based programs. Future well-designed, long-term studies are warranted to confirm these results and to determine whether integration of traditional medicine into clinical practice and public health strategies could contribute to dementia prevention.

## Data Availability

The data analyzed in this study is subject to the following licenses/restrictions: the raw data supporting the conclusion of this article is available from the corresponding author, upon reasonable request. Requests to access these datasets should be directed to S-YC, lovepwr@khu.ac.kr.
