# Supplementary material for: Herbal medicine and acupuncture for mild cognitive impairment: a retrospective study of 2,242 for older adults in Republic of Korea
Source: Front Neurol. 2025 Oct 29;16:1628794. doi: 10.3389/fneur.2025.1628794 (PMC12605537; doi:10.3389/fneur.2025.1628794)
Supplement: Supplementary file 1 [file Table_1.docx]

Supplementary Material

**Supplementary Table 1.** Compositions of herbal formulas

| **Herbal formula** | **Ingredients (weight of each dried herb for one adult for one day)** |
| --- | --- |
| *Modified Guibi-tang*  (加味归脾汤) | Angelica gigas Nakai (Angelicae Gigantis Radix) 7.5 g, Dimocarpus longan Lour. (Longanae Arillus) 7.5 g, Ziziphus jujuba Mill. (Zizyphi Semen) 7.5 g, Polygala tenuifolia Willd. (Polygalae Radix) 7.5 g, Panax ginseng C.A.Mey. (Ginseng Radix) 7.5 g, Astragalus mongholicus Bunge (Astragali Radix) 7.5 g, Atractylodes macrocephala Koidz. (Atractylodis Rhizoma) 7.5 g, Poria cocos Wolf cum Pinus spp. (Poria Sclertum Cum Pini Radix) 7.5 g, Dolomiaea costus (Falc.) Kasana & A.K.Pandey. (Aucklandiae Radix) 3.76 g, Glycyrrhiza uralensis Fisch. ex DC. (Glycyrrhizae Radix et Rhizoma) 2.26 g, Zingiber officinale Roscoe (Zingiberis Rhizoma Crudus) 12.5 g, Ziziphus jujuba Mill. (Zizyphi Fructus) 7.5 g, Paeonia × suffruticosa Andrews (Moutan Cortex Radicis) 7.5 g, Bupleurum chinense DC. (Bupleuri Radix) 7.5 g, Gardenia jasminoides J.Ellis (Gardeniae Fructus) 7.5 g. |
| *Guibi-tang*  (归脾汤) | Angelica gigas Nakai (Angelicae Gigantis Radix) 7.5 g, Dimocarpus longan Lour. (Longanae Arillus) 7.5 g, Ziziphus jujuba Mill. (Zizyphi Semen) 7.5 g, Polygala tenuifolia Willd. (Polygalae Radix) 7.5 g, Panax ginseng C.A.Mey. (Ginseng Radix) 7.5 g, Astragalus mongholicus Bunge (Astragali Radix) 7.5 g, Atractylodes macrocephala Koidz. (Atractylodis Rhizoma) 7.5 g, Poria cocos Wolf cum Pinus spp. (Poria Sclertum Cum Pini Radix) 7.5 g, Dolomiaea costus (Falc.) Kasana & A.K.Pandey. (Aucklandiae Radix) 3.76 g, Glycyrrhiza uralensis Fisch. ex DC. (Glycyrrhizae Radix et Rhizoma) 2.26 g, Zingiber officinale Roscoe (Zingiberis Rhizoma Crudus) 12.5 g, Ziziphus jujuba Mill. (Zizyphi Fructus) 7.5 g. |
| *Yukmijihwang-tang*  (六味地黄汤) | Rehmannia glutinosa (Gaertn.) Libosch. ex DC. (Rehmanniae Radix Preparata) 16 g, Dioscorea oppositifolia L. (Dioscoreae Rhizoma) 8 g, Cornus officinalis Siebold & Zucc. (Corni Fructus) 8 g, Poria cocos Wolf (Poria Sclerotium) 6 g, Paeonia × suffruticosa Andrews (Paeoniae Radicis Cortex) 6 g, Alisma plantago-aquatica subsp. orientale (Sam.) Sam. (Alismatis Rhizoma) 6 g. |
| *Cheonwangbosim-dan*^*^  (天王补心丹) | Rehmannia glutinosa (Gaertn.) Libosch. ex DC. (Rehmanniae Radix Crudus) 30 g, Coptis chinensis Franch. (Coptidis Rhizoma) 15 g, Acorus gramineus Aiton (Acoris Gramineri Rhizoma) 7.5 g, Panax ginseng C.A.Mey. (Ginseng Radix) 3.75 g, Angelica gigas Nakai (Angelicae Gigantis Radix) 3.75 g, Schisandra chinensis (Turcz.) Baill. (Schisandrae Fructus) 3.75 g, Asparagus officinalis L. (Asparagi Tuber) 3.75 g, Thuja occidentalis L. (Thujae Semen) 3.75 g, Ziziphus jujuba Mill. (Zizyphi Semen) 3.75 g, Scrophularia ningpoensis Hemsl. (Scrophulariae Radix) 3.75 g, Salvia miltiorrhiza Bunge (Salviae Miltiorrhizae Radix) 3.75 g, Polygala tenuifolia Willd. (Polygalae Radix) 3.75 g, Poria cocos Wolf cum Pinus spp. (Poria Sclertum Cum Pini Radix) 3.75 g, Liriope spicata Lour. (Liriopis Tuber) 3.75 g, Castanea mollissima Blume (Castaneae Semen) 3.75 g. |
| *Jowiseungcheong-tang*  (调胃升淸汤) | Coix lacryma-jobi L. (Coicis Semen) 16 g, Castanea mollissima Blume (Castaneae Semen) 16 g, Raphanus sativus L. (Raphani Semen) 12 g, Dimocarpus longan Lour. (Longanae Arillus) 12 g, Liriope spicata Lour. (Liriopis Tuber) 8 g, Platycodon grandiflorus (Jacq.) A.DC. (Platycodonis Radix) 8 g, Acorus gramineus Aiton (Acoris Gramineri Rhizoma) 8 g, Thuja occidentalis L. (Thujae Semen) 8 g, Ziziphus jujuba Mill. (Zizyphi Semen) 8 g, Massa medicata fermentata (Massa Medicata Fermentata) 8 g, Ephedra sinica Staf (Ephedrae Herba) 6 g, Schisandra chinensis (Turcz.) Baill. (Schisandrae Fructus) 6 g, Wurfbainia villosa (Lour.) Škorničk. & A.D.Poulsen (Amomi Fructus) 6 g, Polygala tenuifolia Willd. (Polygalae Radix) 6 g. |
| *Ondam-tang*  (温胆汤) | Pinellia ternata (Thunb.) Makino (Pinelliae Tuber) 15 g, Citrus × aurantium L. (Citri Unshius Pericarpium) 15 g, Poria cocos Wolf (Poria Sclerotium) 15 g, Citrus trifoliata L. (Ponciri Fructus Immaturus) 15 g, Phyllostachys edulis (Carriére) J.Houz. (Phyllostachyos Caulis in Taeniam) 7.5 g, Ziziphus jujuba Mill. (Zizyphi Fructus) 6 g, Zingiber officinale Roscoe (Zingiberis Rhizoma Recens) 4 g, Glycyrrhiza uralensis Fisch. ex DC. (Glycyrrhizae Radix et Rhizoma) 3.76 g. |
| *Modified Ukgan-san*  (加味抑肝散) | Pinellia ternata (Thunb.) Makino (Pinelliae Tuber) 10 g, Atractylodes macrocephala Koidz. (Atractylodis Rhizoma Alba) 8 g, Poria cocos Wolf (Poria Sclerotium) 8 g, Cnidium monnieri (L.) Cusson (Cnidii Rhizoma) 6 g, Angelica gigas Nakai (Angelicae Gigantis Radix) 6 g, Uncaria rhynchophylla (Miq.) Miq. (Uncariae Ramulus cum Uncus) 6 g, Citrus × aurantium L. (Citri Unshius Pericarpium) 6 g, Bupleurum chinense DC. (Bupleuri Radix) 4 g, Glycyrrhiza uralensis Fisch. ex DC. (Glycyrrhizae Radix et Rhizoma) 3 g. |
| *Hwanglyeonhaedok-tang*  (黄连解毒汤) | Coptis chinensis Franch. (Coptidis Rhizoma) 9.38 g, Scutellaria baicalensis Georgi (Scutellariae Radix) 9.38 g, Phellodendron amurense Rupr. (Phellodendri Cortex) 9.38 g, Gardenia jasminoides J.Ellis (Gardeniae Fructus) 9.38 g. |
| *** a unique formula only used in Korea | |
