# Supplementary material for: Herbal medicine and acupuncture for mild cognitive impairment: a retrospective study of 2,242 for older adults in Republic of Korea
Source: Front Neurol. 2025 Oct 29;16:1628794. doi: 10.3389/fneur.2025.1628794 (PMC12605537; doi:10.3389/fneur.2025.1628794)
Supplement: Supplementary file 2 [file Table_2.docx]

Supplementary Material

**Supplementary Table 2.** Comparison of cognitive outcomes across major herbal prescriptions (herbal add-on group only)

| **Prescription** | **n** | **Δ CIST  (Mean ± SD)** | **Coefficients  (95% CI)** | **t-value** |
| --- | --- | --- | --- | --- |
| *Guibi-tang* | 637 | 4.09 ± 3.57 | Ref. |  |
| *Modified Guibi-tang* | 810 | 3.94 ± 3.60 | -0.19 (-0.55, 0.17) | -1.038 |
| *Cheonwangbosim-dan* | 157 | 3.96 ± 3.69 | -0.18 (-0.79, 0.43) | -0.588 |
| *Jowiseungcheong-tang* | 99 | 4.41 ± 4.28 | 0.22 (-0.51, 0.96) | -0.594 |
| *Hwanglyeonhaedok-tang* | 14 | 3.57 ± 2.62 | -0.18 (-2.02, 1.66) | -0.194 |
| *Yukmijihwang-tang* | 166 | 3.73 ± 3.76 | -0.19 (-0.78, 0.41) | -0.615 |
| *Ondam-tang* | 104 | 4.26 ± 3.75 | 0.23 (-0.50, 0.95) | 0.612 |
| *Modified Ukgan-san* | 57 | 4.91 ± 4.34 | 0.75 (-0.19, 1.69) | 1.555 |
