# Supplementary material for: Herbal medicine and acupuncture for mild cognitive impairment: a retrospective study of 2,242 for older adults in Republic of Korea
Source: Front Neurol. 2025 Oct 29;16:1628794. doi: 10.3389/fneur.2025.1628794 (PMC12605537; doi:10.3389/fneur.2025.1628794)
Supplement: Supplementary file 3 [file Table_3.docx]

Supplementary Material

**Supplementary Table 3.** Robust regression analysis between two groups after 1:1 propensity score matching

| **Outcomes** | **Coefficients** | **Std. Error** | **t value** | **95% CI** |
| --- | --- | --- | --- | --- |
| CIST | 0.95 | 0.331 | 2.876 | (0.30, 1.60) |
| MoCA | 0.44 | 0.311 | 1.427 | (-0.17, 1.05) |
| GDS | -0.34 | 0.294 | -1.147 | (-0.91, 0.24) |

Abbreviations: CIST, Cognitive Impairment Screening Test; GDS-SF, Geriatric Depression Scale-Short Form; MoCA, Montreal Cognitive Assessment.
