# Supplementary material for: Herbal medicine and acupuncture for mild cognitive impairment: a retrospective study of 2,242 for older adults in Republic of Korea
Source: Front Neurol. 2025 Oct 29;16:1628794. doi: 10.3389/fneur.2025.1628794 (PMC12605537; doi:10.3389/fneur.2025.1628794)
Supplement: Supplementary file 4 [file Image_1.pdf]

Supplementary Material

Supplementary Fig. 1 Overview of KSHPP process and outcome assessment

	Pre	Treatment					Post
**TIMEPOINT**	**Within - 2 weeks**	**4–8 weeks**				**2 weeks**	**Within + 2 weeks**
**ENROLMENT:**							
Eligibility screen	X						
Informed consent	X						
**TREATMENT:**							
Acupuncture		◆——	——	——	——	——◆	
Herbal medicine (decoction)						◆——◆	
**ASSESSMENTS:**							
MoCA	X						X
CIST	X						X
GDS-SF	X						X

Abbreviations: CIST, Cognitive Impairment Screening Test; GDS-SF, Geriatric Depression Scale-Short Form; MoCA, Montreal Cognitive Assessment.
